# Sustained Results in Long-Term Follow-Up of Autologous Chondrocyte Implantation (ACI) for Distal Femur Juvenile Osteochondritis Dissecans (JOCD)

**DOI:** 10.1155/2018/7912975

**Published:** 2018-09-23

**Authors:** Jennifer J. Beck, Dai Sugimoto, Lyle Micheli

**Affiliations:** ^1^Orthopedic Institute for Children/UCLA, Los Angeles, CA, USA; ^2^The Micheli Center for Sports Injury Prevention, Waltham, MA, USA; ^3^Boston Children's Hospital Orthopaedics and Sports Medicine, Boston, MA, USA; ^4^Department of Orthopaedic Surgery, Harvard Medical School, Boston, MA, USA

## Abstract

**Introduction:**

Concern regarding ability of autologous chondrocyte implantation (ACI) to correct for the bone and cartilage pathology of knee juvenile osteochondritis dissecans (JOCD) exists. The purpose of this study was to determine long-term, patient-based outcomes of ACI treatment of JOCD in young patients. Authors hypothesized long-term outcomes are comparable to reported mid-term outcomes.

**Methods:**

A single institution, longitudinal cohort study design combining medical record review and outcome surveys was used. Inclusion criteria included isolated JOCD diagnosis, failed primary healing of operatively treated JOCD, ACI surgery > 5 years ago, and ≤20 years of age at time of ACI.

**Results:**

10/26 eligible patients (38.5%) participated (M: F = 5:5, age at ACI: 18.3 ± 2.5 y, current age: 30.8 ± 5.1 y, and current BMI: 24.6 ± 2.1). Follow-up was 12.0 ± 4.5 y. Lesion size at ACI was 9.1 ± 1.9 cm^2^. Femoral condyle location was medial = 6 and lateral = 4. All required treatment at some point for knee symptoms after ACI. During the past one year, four patients required treatment. Patient reported outcome scores at 12 years following ACI were IKDC score: 73.0 ± 3.6, KOOS scores including pain [88.7 ± 2.3], symptoms [78.2 ± 4.6], activity of daily living [94.7 ± 1.9], function, sports, and recreational activities [73.0 ± 5.3], and quality of life [57.5 ± 5.8], and Modified Cincinnati Knee Rating score was 77.9 ± 4.1. A moderate to good relationship was found between KOOS symptoms and BMI and lesion size. Function, sports, and recreational activities of the KOOS were greater in participants who had ≤1 lesion prior to ACI procedures (p = 0.044).

**Conclusion:**

This study of ACI treatment of knee JOCD patients confirms sustained, long-term results. Number of lesions prior to ACI procedure influenced status of function, sports, and recreational activities.

## 1. Introduction

Osteochondritis dissecans (OCD) is a focal alteration in subchondral bone risking disruption of overlying articular cartilage and possibly leading to premature osteoarthritis [1]. This condition is thought of as a primarily bone pathology with secondary effects on the overlying cartilage. The patients' symptoms, skeletal maturity, and radiographic staging guide treatment. Commonly found in the knee, plain radiographs are used reliably for diagnosis, staging [2], and management. Advanced imaging, such as MRI, is often used in conjunction with radiographs and physical exam for staging and surgical planning though inconsistencies in protocols and findings limit its clinical utility [3–5].

Increasingly being diagnosed in skeletally immature athletes, juvenile osteochondritis dissecans (JOCD) can be problematic to manage in young, skeletally immature, highly active patients. Histologic cartilage studies have determined age of 20 years as a transition period between adult and juvenile cartilage [6] defining JOCD as patients less than 20 years of age at time of presentation. Male patients have up to 4 times the risk of JOCD compared to female while patients aged 12-19 years have 3 times the risk in comparison with 6-11-year-old patients [7]. Approximately 30% of patients have bilateral lesions [8]. Primary surgical intervention of the knee JOCD is indicated for failure of nonoperative management, advanced stage, or advanced age [9] and consists of microfracture, drilling, or fixation depending on the stage [10].

Salvage options after failure of knee JOCD primary surgical intervention are controversial and include autograft and allograft options. Autologous Chondrocyte Implantation (ACI) is one surgical option for failed JOCD healing. Concern regarding ability of ACI to correct for the bone and cartilage pathology of knee JOCD exists. Reports of bone edema after matrix-assisted autologous chondrocyte transplantation (MACT) did not correlate with a poorer outcome [11]. Favorable factors for ACI success are patients who are younger, have higher preoperative symptom scores, have a single lesion on the trochlea or lateral femoral condyle, and had less than 2 previous procedures on the knee [12].

A trend towards improvement in JOCD treatment results of ACI over osteochondral autologous transplantation, synthetic scaffolds, and other autologous or allograft reconstruction techniques exists [13]. Outcomes of adult OCD lesions treated by ACI are impacted by size of the OCD lesion with success rates lower than JOCD patients [14, 15]. Mid-term results for JOCD have been promising with improved outcome scores reported [14, 16, 17] with earlier age at implantation improving surgical success. Mid-term result failure in adult OCD is estimated around 20% [18] versus 3% in JOCD [16].

Ten-year follow-up of adult OCD lesions treated with ACI found 7% failure with another 9% requiring a graft related procedure within the first year of implantation, no patient reached their preinjury activity level, and nearly half of patients had radiographic evidence of osteoarthritis [19]. Long-term outcomes have not been reported for ACI in knee JOCD, especially in a physically active, youth population. Therefore, the purpose of this study is to examine long-term outcomes of ACI on young patients who failed primary surgical treatment of knee JOCD. Authors hypothesize long-term outcomes of ACI are comparable to previously reported mid-term outcomes [16].

## 2. Materials and Methods

### 2.1. Study Methodology

A single institution, retrospective, longitudinal cohort study design combining medical record review and outcome surveys was developed. Prior to study initiation, IRB approval was obtained.

Patients were included if they had isolated distal femur JOCD diagnosis, failed primary healing of operatively treated JOCD, ACI surgery > 5 years ago, and < 20 years of age at time of ACI. Twenty-six patients were identified by record review. Ten patients were able to be contacted and consented for study participation. All patients were > 18 years of age and able to provide institution approved, informed consent. One patient was contacted and refused participation in this study. Fifteen patients were unable to be contacted despite multiple attempts via mail, phone calls, and email methods. Therefore, a total of 10 out of 26 eligible patients (38%) participated.

International Knee Documentation Committee (IKDC), Knee injury and Osteoarthritis Outcome Score (KOOS), and Modified Cincinnati Knee Rating outcome scores were collected. A uniquely created survey consisting of questions regarding a history of treatments after ACI surgery was also completed by study participants (Appendix).

Scores from the IKDC, KOOS, Modified Cincinnati Rating outcome, and the survey created for this investigation were entered into a database for data analysis. Medical records were thoroughly reviewed for the ten patients enrolled in the study. During medical record review, diagnosis, history of procedures prior to ACI, lesion location, lesion size, age at implantation, concomitant procedure performed during ACI, ACI procedure details, postoperative complications, and contralateral knee surgeries were extracted and documented to confirm JOCD history and status following ACI.

### 2.2. Statistical Analysis

Baseline characteristics of patients including current age, height, weight, and body mass index (BMI) along with history of JOCD and detailed information of orthopedic procedures prior to ACI were examined. Also, patients' outcome surveys including IKDC, KOOS, and Modified Cincinnati Knee Rating Scale scores were analyzed by a descriptive statistics using mean, standard deviation, median, and ranges. Frequency of treatment history since ACI surgery and within the last year was examined by count and percentages (%). Correlation coefficient analysis was used to find an association between the three patients' outcome scales and lesion size, BMI, and age at ACI implantation. Correlation coefficient values (r) were selected to determine strength of the association, and the following criteria were used: little or no relationship (r = 0.00 – 0.25), fair (r = 0.26-0.50), moderate to good (r = 0.51-0.75), good to excellent (r = 0.76-1.00) [20]. The patients' outcome surveys were compared based on sex (male versus females), ACI location (medial versus lateral), and number of lesions before ACI (≤1 versus >1). Because of the small sample size, nonparametric t-test, Mann–Whitney U test, was employed to investigate whether there is a significant difference in the IKDC, KOOS, and Modified Cincinnati Knee Rating Scales. Cohen's d was also used to express effect size along with the Mann–Whitney U test. The following Cohen's d was used to express effect sizes: 0.2-0.3 was small, 0.5 was medium, and ≥0.8 was large. Statistical significance of 0.05 was used. Statistical Package for the Social Sciences (version 21, SPSS Inc., Armonk, NY, USA) was used for all analyses.

## 3. Results

### 3.1. Baseline Characteristics

Baseline information of physical characteristic, JOCD history, and orthopedic procedures for JOCD prior to ACI were determined for the ten patients enrolled in this study (Table 1). Equal numbers of male and female patients were included (M: F = 5:5). Patients were on average 18 years old (range 15-22 years) at the time of ACI (Table 1(a)). Mean follow-up duration after ACI was 12.0 ± 4.5 years. The JOCD lesions included six medial femoral condyle and four lateral femoral condyle locations. Eight patients had a periosteal covering, one a porcine mucosa covering, and one synthetic patch covering. These lesions were isolated knee pathology except one case of lateral femoral condyle OCD associated with unstable lateral discoid meniscus.

Prior to ACI, surgical treatment included an average of 2.5 surgical procedures (range 1-5, Table 1(a)) with average of 4.4 years (range, 1-11) from initial surgical treatment of OCD to ACI. Only one patient had one surgery prior to ACI implantation with the rest of the patients having 2+ procedures (Table 1(b)). All patients underwent marrow stimulation and lesion debridement at some points prior to ACI, typically at the time of ACI biopsy. There was less than one year between ACI biopsy and implantation in every patient.

### 3.2. Patient Outcomes

IKDC, KOOS, and Modified Cincinnati Knee Rating Scales scores at final follow-up were calculated (Table 2). All patients required treatment at some point for knee symptoms after ACI. Patient reported treatments in the last year and since ACI were described (Table 3).

The effects of gender, lesion size, BMI, and lesion location were compared with outcome scores (Table 4). Moderate to good correlation was found between BMI and lesion size with KOOS symptoms, though these did not reach statistical significance. Fair or little correlation was found between the other outcome scores and lesion size, BMI, and age at ACI implantation. There were no significant differences between the three outcome scales and sex (female versus male, Table 5) or lesion location (medial versus lateral, Table 6). However, statistical significance was detected by a comparison of number of lesions prior to ACI (≤1 versus >1, Table 7). The function, sports, and recreational activities of the KOOS score in ACI patients ≤1 lesion were significantly greater than those of ACI patients with > 1 (p = 0.044, Table 7). Also, nearly large effect size was found in the function, sports, and recreational activities of the KOOS score in ACI patients with >1 lesion compared to ACI patients with ≤1 (Cohen's d = 0.716, Table 7). Also, greater than moderate effect sizes were recorded in symptoms in KOOS (Cohen's d = 0.637, Table 7) and Modified Cincinnati Knee Rating Scales (Cohen's d = 0.525, Table 7) among ACI patients with ≤ 1 lesion relative to ACI patients with >1.

### 3.3. Subsequent Operations

Retrospective chart review found four patients underwent surgery after ACI during the follow-up period. Three patients were treated by the same senior, primary surgeon at the same institution where they initially received treatment. Two of these three patients underwent arthroscopic loose body excision with specific documentation of an intact ACI graft at six and thirteen years after ACI. The other patient required two arthroscopies for loose body excision, microfracture of the lesion happening four and seven years after implantation. Prior to ACI, this patient had associated symptomatic lateral discoid menisci bilaterally, requiring surgical treatment. Additionally, this patient required tibial bone graft to augment the open reduction, internal fixation of the OCD fragment before going on to ACI for failure of this treatment. The fourth surgical patient was treated at another institution undergoing repeat arthroscopy and ACI resection with reports of ACI “failure” and loose bodies five years after initial ACI implantation. These last two cases can be defined as ACI failure, which was a rate of 20% at an average of twelve years.

In addition to ipsilateral reoperation, chart review showed two patients had contralateral knee surgery for OCD lesions, both requiring a second surgery for excision and microfracture.

## 4. Discussion

To our knowledge, this is the first long-term study of ACI treatment of knee JOCD patients using patients' reported outcome measures: KOOS, IKDC, Modified Cincinnati Knee Rating scores, and treatment history survey. Compared to previously reported literature [16], outcome scores observed in this study showed sustained improvement through twelve years. Thus our hypothesis was confirmed that ACI is a valid long-term treatment option for JOCD. Lesion size and BMI had only moderately correlated with KOOS symptom score and had poor correlation with other patient-based outcomes. Sex and lesion location also did not show any statistical differences with long-term outcome measures. However, number of lesions prior to ACI procedures demonstrated interesting findings. ACI patients with ≤1 lesion showed greater than moderate effect sizes on KOOS symptoms, KOOS-function, sports, and recreational activities, and Modified Cincinnati Knee Rating Scales compared to ACI patients with >1 lesion. Although statistical significance value was observed in only one variable (KOOS-function, sports, and recreational activities), all components of the three outcome surveys were more favorable for ≤1 lesion. Thus, number of lesions before ACI procedures may potentially influence a long-term outcome of ACI.

Microfracture results, as the original treatment technique for OCD, are the baseline to which other techniques are compared. Several studies have compared osteochondral autologous transplantation to microfracture with results showing improved outcomes for a sustained period of time of osteochondral autologous transplantation over microfracture [21, 22]. Athletic patients had a higher return to activities and results were improved when patients were treated at a younger age [19]. In the only comparison study involving pediatric patients with an average age of 14 years (12 to 18), 91% had good to excellent outcomes with osteochondral autologous transplantation and only 56% with microfracture [23].

Histological studies that examined developmental changes of focal cartilage lesions over four years after ACI reported characteristics of articular cartilage over hyaline cartilage [24, 25]. Additionally, histologic studies comparing ACI to other cartilage restoration techniques showed ACI had similar articular cartilage architecture to osteochondral autografts and improved articular cartilage architecture to microfracture [26]. Given increasing life expectancy and increased activity levels, adolescents with articular cartilage lesions require the cartilage restoration procedure that most closely resembles native articular cartilage.

However, the majority of cartilage restoration studies focused on adult patients. ACI outcomes for isolated full-thickness chondral lesions have been reported up to twenty years after index surgery. Improvement in outcome scores has been sustained through this time period [12, 15, 18, 19, 24, 27–31]. Nearly half of adults who underwent ACI procedures reduced their sports intensity and only 40% maintained “similar” training on average five years after surgery [32]. Although the results did not exactly match with clinical outcomes, magnetic resonance imaging (MRI) between 9 and 18 years after implantation indicated the tissue quality of the repair was similar to surrounding articular cartilage with restoration of the defect area in the majority of patients [33]. These outcomes are considered to defer from ACI outcomes in JOCD due to the lack of underlying bone pathology and involvement.

Recent systematic review of ACI in the adolescent knee followed for 52 months (12 to 74 months) showed an average improvement of outcome scores near 40% with graft hypertrophy being the most common complication [34]. Adolescent patients undergoing ACI for OCD have 96% good to excellent outcomes at the mid-term [17]. Similarly, 96% of patients also returned to high impact sports and 60% returned to a level equal to or higher than prior to their knee injury [17]. Improved results were seen in patients with shorter duration of symptoms (<12 months) and fewer surgeries [17, 34]. Looking at soccer specifically, younger patients, isolated lesions, shorter duration of symptoms, and lower number of surgeries correlated with improved rate of returning to soccer [35]. Our study was underpowered to be able to compare previous surgery numbers with outcomes. However, these previous studies demonstrated prompt treatment of JOCDs is paramount given their improved results.

Compared to the results of this study, a shorter follow-up study employed a similar cohort of JOCD patients with several years of follow-up duration (minimum of two years and mean follow-up time of four years) [16]. The study reported Modified Cincinnati Knee Rating score of 72 with an overall failure rate of 3% while the current study had a score of 78 and a failure rate of 20%. Nineteen percent of patients had undergone a subsequent surgical procedure by four-year follow-up in the previous study while the current study showed an increased surgical rate to 40% by twelve-year follow-up. Graft hypertrophy and graft failure were the common causes of need for subsequent surgery. The same senior author was involved in both studies and there is a high likelihood that patients enrolled in our study were also involved in the earlier study. However, previous databases were not available to confirm this suspicion.

Rosa et al. [36] published a similar study to the current study and concluded that ACI is an effective, durable solution for full-thickness cartilage and osteochondral lesions of the knee in young patients. With similar 12-year follow-up, 15 patients with average age of 21 years (range 13-45 years, standard deviation of 8.9 years) were treated with ACI for full-thickness cartilage and osteochondral lesions. Only seven patients were OCD patients and none of their patients had prior surgeries to treat their lesions. Average lesion size was slightly smaller than the current study (5.08 ± 2.01 versus 9.1 ± 1.9 cm^2^). Similarly, a two-step ACI technique was performed in both studies.

The majority of our results were comparable in these two similar studies. Failure of ACI has been previously defined [16] as subsequent operation that either (1) necessitated removal of the graft, (2) confirmed a loss of defect fill, or (3) violated the subchondral bone (e.g., abrasion chondroplasty, microfracture, drilling, unicompartmental arthroplasty, or total knee arthroplasty). Both studies recorded two failures resulting in graft resection, though Rosa et al. did not define which patients, isolated cartilage or osteochondral lesions, failed. Outcome scores were similar between Rosa et al. and the current study with IKDC (76.3 versus 73.0), KOOS Pain (79.6 versus 88.7), symptoms (76.4 versus 78.2), activities of daily living (85.1 versus 94.7), and sports (70.3 versus 73.0). Despite these similar scores, Rosa et al. had a higher knee quality of life KOOS score of 74.2 compared to 57.5 documented in this study. Despite high scores on the rest of the outcome measures, speculation as to the lower knee quality of life in the current study is likely related to activity expectations of a younger population and their frustration with any hindrance in activity.

In addition to clinical outcomes, Rosa et al. were able to obtain MRI images around 12 years after surgery on a majority of their patients. Using the Magnetic Resonance Observation of Cartilage Repair Tissue (MOCART) system, the authors detected a significant decrease in MOCART score from short- to long-term follow-up with significant correlation with degree of defect repair and pain KOOS scores [36].

In 2012, the AAOS announced Clinical Practice Guidelines of the diagnosis and treatment of OCD [37]. Statements 8 and 12 stated that the work group is unable to recommend for or against a specific cartilage repair technique in symptomatic cases among skeletally immature or mature patients. The strength of recommendation of these particular statements was “inconclusive”. This work group, however, had “consensus” level recommendation for offering surgery to skeletally mature or immature patients with symptomatic OCDs despite limited literature. This current study may help providing scientifically quality evidence and clinically valuable knowledge into long-term outcomes of JOCD treatment with ACI.

In 2016, the German Society of Orthopedics and Trauma (DGOU) work group on “Clinical Tissue Regeneration” provided guidelines on the use of ACI for knee cartilage defects [38]. Specified indications for ACI in the knee joint to include full-thickness, symptomatic cartilage defects of OCD stages 3 and 4 per ICRS-OCD, possibly in combination with subchondral reconstruction with use in children and adolescents. The group agreed that effect of subchondral edema is largely unknown due to limited available research as only one paper published on the topic concluded they were unable to find correlation between edema and outcomes [11]. Successful subchondral reconstruction has been described with subchondral defects greater than 3 mm undergoing one- or two-stage subchondral reconstruction [30]. Though the current study was not designed to comment on subchondral edema or subchondral reconstruction, our long-term results showed that knee JOCD patients can be successfully treated with ACI.

While ACI is a viable option for cartilage defects of the knee, excision/debridement, bone marrow stimulation, and allograft or autograft osteocartilaginous reconstructions are also available options for surgeons. Mundi et al. [39] published a meta-analysis with systematic review of Level 1 studies of cartilage restoration techniques of the knee. Synthesizing 12 studies completed in Europe on adult patients with variable pathology underwent, meta-analysis indicated no difference between outcomes of ACI and microfracture at 2 years despite majority of individual studies stating improvement of ACI outcomes over microfracture. Also, another limitation of this meta-analysis was not able to analyze comparison of ACI versus osteochondral autograft transplantation due to poor study methodologies and data collections. Therefore, their findings concluded an inability to determine a single best technique for cartilage restoration.

Only two studies have compared ACI versus other surgical treatment options for OCD. A 3-year observational, adult cohort showed ACI had improved knee function scores and greater relief from pain and swelling than debridement [40]. A prospective, randomized study comparing ACI versus mosaicplasty in adults demonstrated superiority of ACI over mosaicplasty with limited 19-month outcome being a main weakness of the study [41]. The relatively short duration of follow-up duration and small sample size of these studies are common limitations. With poor long-term clinical outcomes [42, 43] and evidence of early osteoarthritic changes on radiographs [44], JOCD fragment excision and debridement are less than desirable surgical techniques. Long-term results of osteochondral allografts [45, 46] and autografts [47, 48] indicated promise, but must be used with care in skeletally immature patients and near open physes of the distal femur. In short, surgeons who are planning to conduct future studies need to consider improving study methodologies and outcomes in order to determine the best treatment option for the individual patients, especially skeletally immature patients.

The senior author of this study has noted that most OCD lesions that fail to heal and have undergone fragment removal have necrotic bone at the base of the lesion. Ideally, vascularity must be restored to this base before ACI is attempted. This is usually best done by patterned drilling of the lesion with a .045 smooth pin at least six months prior to implant. MRI can be used to determine the depth required to bypass the necrotic bone. Using microfracture awls are not advised due to the risk of insufficient depth of penetration.

### 4.1. Limitation

Weaknesses of our study include small sample size, which limits generalizability of the current data. Nonsignificant findings by sex and lesion size analysis may be due to the small sample sizes. In addition, clinical or radiographic follow-up at final outcome point would have assisted understanding current status of ACI on patients' perception. At the time of ACI in our patient cohort, this surgical technique was still being developed and validated and therefore was not widely completed accounting for our small sample size. This small sample likely represents some of the first patients undergoing ACI for failed surgical treatment of JOCD. These patients had transitioned from teenage to adult life often moving away from the institution where their ACI was completed and therefore were unable to be examined and evaluated at final outcome.

## 5. Conclusion

The current study aimed to define long-term follow-up of JOCD patients undergoing ACI. The outcome data from patients' records and surveys indicated the ACI can be a viable treatment through twelve years despite the high need for continued treatment. The analysis showed a moderate to good relationship was found between KOOS symptoms and both BMI and lesion size while sex and lesion location had no effect on outcome scores. Additionally, number of lesions before ACI procedures may be an important factor for a long-term effect. In order to confirm the study outcome of this study, future study needs to have a bigger data sample size.

## Figures and Tables

**Table:** (a) Study participant information (N = 10)

	**Mean ± SD**	**Median**	**Range**
**Physical characteristics**			
Current Age (years)	30.8 ± 1.6	31.3	23.0-37.7
Height (cm)	1.80 ± 0.04	1.78	1.60-1.90
Weight (kg)	77.9 ± 4.6	78.9	55.8-105.0
Body Mass Index	24.6 ± 0.67	24.7	21.1-28.9

**JOCD history**			
Age at Time of ACI	18.3 ± 0.19	18.0	15.0-22.0
Lesion Size at Time of ACI	9.1 ± 1.9	7.4	2.3-24.0
Number of Lesions	1.2 ± 0.4	1.0	1.0-2.0
Number of Surgeries before ACI	2.5 ± 1.3	2.0	1.0-5.0
Number of Surgeries after ACI	0.6 ± 0.7	0.5	0.0-2.0

**Table:** (b) Surgical history prior to ACI

Orthopaedic Procedure Prior to ACI	(N)
Isolated Marrow Stimulation	8

Fragment Fixation	4

Bone Graft/Removal of Hardware	1

Excision of fragment/loose bodies	4

Removal of Hardware	2

Other/Unknown	6

Total Procedures	25

**Table 2:** Outcome of IKDC, KOOS, and Modified Cincinnati Knee Rating Scores.

	**Mean ± SD**	**Median**	**Range**
**IKDC**			

Total Score	73.0 ± 3.6	70	66.0-97.0

Symptoms			

Highest level of activity without pain	2.6 ± 0.3	2.5	2.0-4.0

Pain in the past 4 weeks	7.1 ± 0.7	7.5	4.0-10.0

Severity of pain	6.5 ± 1.1	8.0	2.0-10.0

Stiffness or swelling in the past 4 weeks	3.4 ± 0.2	3.0	3.0-4.0

Highest level of activity without swelling	2.9 ± 0.4	3.0	1.0-4.0

Locking or catching in the past 4 weeks	0.5 ± 0.2	0.5	0.0-1.0

Highest level of activity without giving way	2.9 ± 0.4	3.0	1.0-4.0

Sports Activity			

Highest level of activity on a regular basis	2.4 ± 0.3	2.0	1.0-4.0

Ability to perform			

Go up stairs	3.6 ± 0.2	4.0	3.0-4.0

Go down stairs	3.5 ± 0.3	4.0	2.0-4.0

Kneel on the front your knee	2.4 ± 0.3	2.0	1.0-4.0

Squat	2.6 ± 0.4	2.5	1.0-4.0

Sit with your knee bent	3.6 ± 0.3	4.0	2.0-4.0

Rise from a chair	3.7 ± 0.2	4.0	3.0-4.0

Run straight ahead	3.4 ± 0.3	4.0	2.0-4.0

Jump and land on your involved leg	2.9 ± 0.2	3.0	2.0-4.0

Stop and start quickly	3.3 ± 0.3	3.0	2.0-4.0

Function			

0-10 scale prior to knee injury	7.5 ± 1.3	10.0	1.0-10.0

0-10 scale based on current function	8.3 ± 0.4	8.0	7.0-10.0

**KOOS**			

Symptoms	78.2 ± 4.6	73.2	60.7-100.0

Pain	88.7 ± 2.3	91.7	77.8- 97.2

Function, activity of daily living	94.7 ± 1.9	97.8	83.8-100.0

Function, sports and recreational activities	73.0 ± 5.3	72.5	45.0-100.0

Quality of life	57.5 ± 5.8	59.4	25.0- 81.3

**Modified Cincinnati Knee Rating Scales**			

Total Score	77.9 ± 4.1	77.0	60.0-100.0

Sign/Symptoms Questions			

Pain Intensities	13.3 ± 1.2	12.0	8.0-20.0

Swelling	8.2 ± 0.52	8.0	6.0-10.0

Giving way	16.4 ± 1.4	20.0	8.0-20.0

Overall Activity Level	15.1 ± 1.1	16.0	12.0-20.0

Walking	9.6± 0.3	10.0	8.0-10.0

Stairs	8.9 ± 0.4	8.0	8.0-10.0

Running Activities	3.1 ± 0.5	3.0	0.0-5.0

Jumping and Twisting	3.2 ± 0.5	4.0	0.0-5.0

**Table 3:** Description of treatment history.

**Have you had any treatments on ** **your knee since ACI surgery? **	**Frequency (**%**)**
Yes	10/10 (100%)

No	0/10 (0.0%)

If yes, what type of treatments have you had?	

Physical Therapy	8/10 (80.0%)

Brace	5/10 (50.0%)

Steroid Injection	0/10 (0.0%)

Visco Injection	1/10 (10.0%)

Activity Modification	6/10 (60.0%)

Work Restrictions	1/10 (10.0%)

Oral Medications	1/10 (10.0%)

Surgical Intervention	4/10 (40.0%)

**Have you had any treatments on ** **your knee during last year?**	

Yes	4/10 (40.0%)

No	6/10 (60.0%)

If yes, what type of treatments have you had in last year?	

Physical Therapy	0/4 (0.0%)

Brace	2/4 (50.0%)

Steroid Injection	3/4 (75.0%)

Visco Injection	2/4 (50.0%)

Activity Modification	0/4 (75.0%)

Work Restrictions	3/4 (75.0%)

Oral Medications	1/4 (25.0%)

Surgical Intervention	1/4 (25.0%)

**Table 4:** Correlation coefficient analysis of the patients' outcome scores, lesion size, BMI, and age at ACI implantation.

	**Lesion Size**	**BMI**	**Age at ACI Implantation**
**IKDC Total Score **	- 0.29	- 0.28	- 0.08
**KOOS Symptoms**	- 0.51^*∗*^	- 0.51^*∗*^	0.32
**KOOS Pain**	0.04	- 0.49	- 0.03
**KOOS Function, activity of daily living **	- 0.02	0.01	- 0.09
**KOOS Function, sports and recreational activities**	- 0.30	- 0.18	0.23
**KOOS Quality of life **	- 0.12	- 0.06	0.22
**Modified Cincinnati Knee Rating Total Scores**	- 0.01	0.40	- 0.36

Values were expressed by correlation coefficient value r. ^*∗*^Two variables: moderate to good relationships were found between KOOS symptoms and lesion size (p = .135) and KOOS symptoms and BMI (p = .133), but were nonsignificant.

**Table 5:** Comparison of the patients' reported outcome scores by sex.

	**Females (N = 5)**	**Males (N = 5)**	**P-values**	**Effect Size**
**IKDC**				
Total Score	69.4 ± 5.5	74.2 ± 13.0	0.841	0.234

**KOOS**				
Symptoms	82.9 ± 13.0	73.6 ± 15.7	0.222	0.307
Pain	89.0 ± 6.3	88.3 ± 8.7	0.840	0.046
Function, activity of daily living	96.8 ± 4.9	92.7 ± 6.8	0.421	0.327
Function, sports and recreational activities	80.0 ± 10.0	66.0 ± 20.4	0.151	0.399
Quality of life	61.3 ± 16.8	53.8 ± 21.0	0.690	0.193

**Modified Cincinnati Knee Rating Scales**				
Total Score	77.4 ± 12.6	80.0 ± 12.5	0.972	0.103

**Table 6:** Comparison of the patients' reported outcome scores by OCD location.

	**Medial (N = 6)**	**Lateral (N = 4)**	**P-values**	**Effect Size**
**IKDC**				
Total Score	69.5 ± 4.8	75.3 ± 14.8	0.914	0.255

**KOOS**				
Symptoms	75.0 ± 13.6	83.0 ± 16.3	0.476	0.257
Pain	87.6 ± 6.6	90.3 ± 8.6	0.610	0.173
Function, activity of daily living	95.3 ± 5.6	93.8 ± 7.3	0.762	0.115
Function, sports and recreational activities	68.3 ± 14.0	80.0 ± 20.4	0.476	0.292
Quality of life	62.5 ± 13.1	50.0 ± 24.5	0.476	0.303

**Modified Cincinnati Knee Rating Scales**				
Total Score	77.8 ± 9.4	80.0 ± 16.5	0.998	0.082

**Table 7:** Comparison of the patients' reported outcome scores by number of OCD lesions before ACI.

	**≤ 1 OCD Lesion (N = 8)**	**> 1 OCD Lesion (N = 2)**	**P-values**	**Effect Size**
**IKDC**				
Total Score	72.8 ± 10.8	68.0 ± 1.4	0.711	0.298

**KOOS**				
Symptoms	81.7 ± 14.0	64.3 ± 5.1	0.089	0.637^†^
Pain	89.0 ± 7.2	87.5 ± 9.8	0.889	0.087
Function, activity of daily living	95.0 ± 6.2	93.4 ± 7.3	0.711	0.117
Function, sports and recreational activities	78.1 ± 14.1	52.5 ± 10.6	0.044^*∗*^	0.716^†^
Quality of life	58.6 ± 20.0	53.1 ± 13.3	0.711	0.160

**Modified Cincinnati Knee Rating Scales**				
Total Score	80.9 ± 12.4	70.0 ± 1.4	0.400	0.525^†^

^*∗*^Statistically significant differences were noted with p < 0.05. ^†^Moderate effect size was found.

**Table 8:** 

	**What was the name of the procedure? **	**What date was the surgery (month/year)? **	**Who was the surgeon? **
1.			
2.			
3.			
4.			
5.			

## Data Availability

The data used to support the findings of this study are available from the corresponding author upon request.

## Appendix

### Uniquely Created Survey of Treatments Rendered since Time of ACI Procedure

Thank you for agreeing to participate in our study. We would like to ask you some additional follow up questions about how you are doing. Please answer them as best you can.

Name: —

Biological Sex: —

Height: —

Weight: —

Current Occupation: —

(1) Have you required any treatment for your knee since your ACI surgery? □ Yes □ No

**If Yes**,

Please mark all non-operative treatments required since your ACI:

  □ PT
  □ Brace
  □ Steroid Injection
  □ Visco injection
  □ Activity modification
  □ Work restrictions
  □ Oral medications
  □ Other: —

Please mark all non-operative treatments required in the** last year**:

  □ PT
  □ Brace
  □ Steroid Injection
  □ Visco injection
  □ Activity modification
  □ Work restrictions
  □ Oral medications
  □ Other: —

(2) Please mark all imaging studies of your knee you've had in the past five years:

  □ Xray
  □ MRI
  □ CT
  □ Bone scan

(4) Where were they completed?—

(5) Please list any additional surgeries to your knee after having ACI performed by Dr. Micheli? See Table 8

(6) Can we contact you if we have further questions regarding your treatment history? □ Yes □ No

## References

[1] Edmonds E. W., Shea K. G. (2013). Osteochondritis dissecans: editorial comment. *Clinical Orthopaedics and Related Research*.

[2] Wall E. J., Polousky J. D., Shea K. G. (2015). Novel radiographic feature classification of knee osteochondritis dissecans: A multicenter reliability study. *The American Journal of Sports Medicine*.

[3] Ellermann J. M., Donald B., Rohr S. (2016). Magnetic Resonance Imaging of Osteochondritis Dissecans: Validation Study for the ICRS Classification System. *Academic Radiology*.

[4] Quatman C. E., Quatman-Yates C. C., Schmitt L. C., Paterno M. V. (2012). The clinical utility and diagnostic performance of MRI for identification and classification of knee osteochondritis dissecans. *The Journal of Bone & Joint Surgery*.

[5] Robbach B. P., Paulus A. C., Niethammer T. R. (2016). Discrepancy between morphological findings in juvenile osteochondritis dissecans (OCD): a comparison of magnetic resonance imaging (MRI) and arthroscopy. *Knee Surgery, Sports Traumatology, Arthroscopy*.

[6] Schmal H., Pestka J. M., Salzmann G., Strohm P. C., Südkamp N. P., Niemeyer P. (2013). Autologous chondrocyte implantation in children and adolescents. *Knee Surgery, Sports Traumatology, Arthroscopy*.

[7] Kessler J. I., Nikizad H., Shea K. G., Jacobs J. C., Bebchuk J. D., Weiss J. M. (2014). The demographics and epidemiology of osteochondritis dissecans of the knee in children and adolescents. *The American Journal of Sports Medicine*.

[8] Cooper T., Boyles A., Samora W. P., Klingele K. E. (2014). Prevalence of bilateral JOCD of the knee and associated risk factors. *Journal of Pediatric Orthopaedics*.

[9] Salci L., Ayeni O., Abouassaly M. (2014). Indications for surgical management of osteochondritis dissecans of the knee in the pediatric population: a systematic review.. *The Journal of Knee Surgery*.

[10] Yellin J. L., Gans I., Carey J. L., Shea K. G., Ganley T. J. (2017). The surgical management of osteochondritis dissecans of the knee in the skeletally immature: A survey of the Pediatric Orthopaedic Society of North America (POSNA) membership. *Journal of Pediatric Orthopaedics*.

[11] Filardo G., Kon E., Di Martino A. (2014). Is the clinical outcome after cartilage treatment affected by subchondral bone edema?. *Knee Surgery, Sports Traumatology, Arthroscopy*.

[12] Kon E., Vannini F., Buda R. (2012). How to Treat Osteochondritis Dissecans of the Knee: Surgical Techniques and New Trends. *The Journal of Bone and Joint Surgery-American Volume*.

[13] Krishnan S. P., Skinner J. A., Bartlett W. (2006). Who is the ideal candidate for autologous chondrocyte implantation?. *The Journal of Bone & Joint Surgery (British Volume)*.

[14] Krishnan S. P., Skinner J. A., Carrington R. W. J., Flanagan A. M., Briggs T. W. R., Bentley G. (2006). Collagen-covered autologous chondrocyte implantation for osteochondritis dissecans of the knee. Two- to seven-year results. *The Journal of Bone & Joint Surgery (British Volume)*.

[15] Pascual-Garrido C., Friel N. A., Kirk S. S. (2017). Midterm Results of Surgical Treatment for Adult Osteochondritis Dissecans of the Knee. *The American Journal of Sports Medicine*.

[16] Micheli L. J., Moseley J. B., Anderson A. F. (2006). Articular cartilage defects of the distal femur in children and adolescents: Treatment with autologous chondrocyte implantation. *Journal of Pediatric Orthopaedics*.

[17] Mithöfer K., Minas T., Peterson L., Yeon H., Micheli L. J. (2005). Functional outcome of knee articular cartilage repair in adolescent athletes. *The American Journal of Sports Medicine*.

[18] Cole B. J., DeBerardino T., Brewster R. (2012). Outcomes of Autologous Chondrocyte Implantation in Study of the Treatment of Articular Repair (STAR) Patients With Osteochondritis Dissecans. *The American Journal of Sports Medicine*.

[19] Martinčič D., Radosavljevič D., Drobnič M. (2014). Ten-year clinical and radiographic outcomes after autologous chondrocyte implantation of femoral condyles. *Knee Surgery, Sports Traumatology, Arthroscopy*.

[20] Portney L. G., Watkins M. P. (2000). *Foundations of clinical research: applications to practice*.

[21] Gudas R., Gudaite A., Pocius A. (2012). Ten-year follow-up of a prospective, randomized clinical study of mosaic osteochondral autologous transplantation versus microfracture for the treatment of osteochondral defects in the knee joint of athletes. *The American Journal of Sports Medicine*.

[22] Gudas R., Stankevičius E., Monastyreckiene E., Pranys D., Kalesinskas R. J. (2006). Osteochondral autologous transplantation versus microfracture for the treatment of articular cartilage defects in the knee joint in athletes. *Knee Surgery, Sports Traumatology, Arthroscopy*.

[23] Gudas R., Simonaityte R., Čekanauskas E., Tamošiunas R. (2009). A prospective, randomized clinical study of osteochondral autologous transplantation versus microfracture for the treatment of osteochondritis dissecans in the knee joint in children. *Journal of Pediatric Orthopaedics*.

[24] Peterson L., Brittberg M., Kiviranta I., Åkerlund E. L., Lindahl A. (2002). Autologous chondrocyte transplantation: biomechanics and long-term durability. *The American Journal of Sports Medicine*.

[25] Peterson L., Minas T., Brittberg M., Nilsson A., Sjögren-Jansson E., Lindahl A. (2000). Two-to 9-year outcome after autologous chondrocyte transplantation of the knee. *Clinical Orthopaedics and Related Research*.

[26] DiBartola A. C., Everhart J. S., Magnussen R. A. (2016). Correlation between histological outcome and surgical cartilage repair technique in the knee: A meta-analysis. *The Knee*.

[27] Beris A. E., Lykissas M. G., Kostas-Agnantis I., Manoudis G. N. (2012). Treatment of full-thickness chondral defects of the knee with autologous chondrocyte implantation: A functional evaluation with long-term follow-up. *The American Journal of Sports Medicine*.

[28] Bhosale A. M., Kuiper J. H., Johnson W. E., Harrison P. E., Richardson J. B. (2017). Midterm to Long-Term Longitudinal Outcome of Autologous Chondrocyte Implantation in the Knee Joint. *The American Journal of Sports Medicine*.

[29] Minas T., Von Keudell A., Bryant T., Gomoll A. H. (2014). The John Insall Award: A Minimum 10-year Outcome Study of Autologous Chondrocyte Implantation. *Clinical Orthopaedics and Related Research*.

[30] Niemeyer P., Porichis S., Steinwachs M. (2014). Long-term outcomes after first-generation autologous chondrocyte implantation for cartilage defects of the knee. *The American Journal of Sports Medicine*.

[31] Peterson L., Vasiliadis H. S., Brittberg M., Lindahl A. (2010). Autologous chondrocyte implantation: a long-term follow-up. *The American Journal of Sports Medicine*.

[32] Pestka J. M., Feucht M. J., Porichis S., Bode G., Südkamp N. P., Niemeyer P. (2016). Return to Sports Activity and Work after Autologous Chondrocyte Implantation of the Knee: Which Factors Influence Outcomes?. *The American Journal of Sports Medicine*.

[33] Vasiliadis H. S., Danielson B., Ljunberg M. (2010). Autologous chondrocyte implantation in cartilage lesions of the knee: long-term evaluation with magnetic resonance imaging and delayed gadolinium-enhanced magnetic resonance imaging technique. *The American Journal of Sports Medicine*.

[34] DiBartola A. C., Wright B. M., Magnussen R. A., Flanigan D. C. (2016). Clinical Outcomes After Autologous Chondrocyte Implantation in Adolescents’ Knees: A Systematic Review. *Arthroscopy - Journal of Arthroscopic and Related Surgery*.

[35] Mithöfer K., Peterson L., Mandelbaum B. R., Minas T. (2005). Articular cartilage repair in soccer players with autologous chondrocyte transplantation: Functional outcome and return to competition. *The American Journal of Sports Medicine*.

[36] Rosa D., Balato G., Ciaramella G., Soscia E., Improta G., Triassi M. (2016). Long-term clinical results and MRI changes after autologous chondrocyte implantation in the knee of young and active middle aged patients. *Journal of Orthopaedics and Traumatology*.

[37] Chambers H. G., Shea K. G., Carey J. L. (2011). AAOS Clinical Practice Guideline: Diagnosis and Treatment of Osteochondritis Dissecans. *American Academy of Orthopaedic Surgeon*.

[38] Niemeyer P., Albrecht D., Andereya S. (2016). Autologous chondrocyte implantation (ACI) for cartilage defects of the knee: a guideline by the working group ‘Clinical Tissue Regeneration’ of the German Society of Orthopaedics and Trauma (DGOU). *The Knee*.

[39] Mundi R., Bedi A., Chow L. (2016). Cartilage Restoration of the Knee. *The American Journal of Sports Medicine*.

[40] Fu F. H., Zurakowski D., Browne J. E. (2005). Autologous chondrocyte implantation versus debridement for treatment of full-thickness chondral defects of the knee: An observational cohort study with 3-year follow-up. *The American Journal of Sports Medicine*.

[41] Bentley G., Biant L. C., Carrington R. W. J. (2003). A prospective, randomised comparison of autologous chondrocyte implantation versus mosaicplasty for osteochondral defects in the knee. *The Journal of Bone & Joint Surgery (British Volume)*.

[42] Michael J. W., Wurth A., Eysel P., König D. P. (2008). Long-term results after operative treatment of osteochondritis dissecans of the knee joint—30 year results. *International Orthopaedics*.

[43] Wright R. W., McLean M., Matava M. J., Shively R. A. (2004). Osteochondritis dissecans of the knee: Long-term results of excision of the fragment. *Clinical Orthopaedics and Related Research*.

[44] Murray J. R. D., Chitnavis J., Dixon P. (2007). Osteochondritis dissecans of the knee; long-term clinical outcome following arthroscopic debridement. *The Knee*.

[45] Lyon R., Nissen C., Liu X. C., Curtin B. (2013). Can fresh osteochondral allografts restore function in juveniles with osteochondritis dissecans of the knee? Knee. *Clinical Orthopaedics and Related Research*.

[46] Raz G., Safir O. A., Backstein D. J., Lee P. T. H., Gross A. E. (2014). Distal femoral fresh osteochondral allografts: Follow-up at a mean of twenty-two years. *Journal of Bone and Joint Surgery - American Volume*.

[47] Sasaki K., Matsumoto T., Matsushita T. (2012). Osteochondral autograft transplantation for juvenile osteochondritis dissecans of the knee: A series of twelve cases. *International Orthopaedics*.

[48] Stone K. R., Pelsis J. R., Crues J. V., Walgenbach A. W., Turek T. J. (2014). Osteochondral grafting for failed knee osteochondritis dissecans repairs. *The Knee*.

